# Outcomes of empiric treatment for pediatric tuberculosis, Kampala, Uganda, 2010–2015

**DOI:** 10.1186/s12889-019-6821-2

**Published:** 2019-04-29

**Authors:** Eric Wobudeya, Devan Jaganath, Moorine Penninah Sekadde, Betty Nsangi, Heather Haq, Adithya Cattamanchi

**Affiliations:** 1 Directorate of Pediatrics & Child Health, Mulago National Referral Hospital, P.O. Box 23491, Kampala, Uganda; 2 Division of Pediatric Infectious Diseases, University of California, 550 16th St. 4th floor, San Francisco, CA 94158 USA; 3 National TB and Leprosy Program (NTLP), Plot 6, Lourdel Road, Nakasero, P. O. Box 7272, Kampala, Uganda; 4 USAID RHITES-EC, University Research Co. LLC, Plot 40, Ntinda II Road, PO Box 28745, Kampala, Uganda; 5 Department of Pediatrics, Baylor College of Medicine, Houston, TX USA; 6 Division of Pulmonary and Critical Care Medicine, University of California, 1001 Potrero Ave, SFGH 5, San Francisco, CA 94110 USA; 7 Center for Vulnerable Populations, Department of Medicine, University of California, San Francisco, USA; 8 Curry International Tuberculosis Center, University of California, San Francisco, USA

**Keywords:** Child, Tuberculosis, Treatment, Outcomes

## Abstract

**Background:**

Childhood tuberculosis (TB) diagnoses often lack microbiologic confirmation and require empiric treatment. Barriers to empiric treatment include concern for poor outcomes and adverse effects. We thus determined the outcomes of empiric TB treatment from a retrospective cohort of children at a national referral hospital in Kampala, Uganda from 2010 to 2015.

**Methods:**

Children were diagnosed clinically and followed through treatment. Demographics, clinical data, outcome and any adverse events were extracted from patient charts. A favorable outcome was defined as a child completing treatment with clinical improvement. We performed logistic regression to assess factors associated with loss to follow up and death.

**Results:**

Of 516 children, median age was 36 months (IQR 15–73), 55% (95% CI 51–60%) were male, and HIV prevalence was 6% (95% CI 4–9%). The majority (*n* = 422, 82, 95% CI 78–85%) had a favorable outcome, with no adverse events that required treatment discontinuation. The most common unfavorable outcomes were loss to follow-up (57/94, 61%) and death (35/94, 37%; overall mortality 7%). In regression analysis, loss to follow up was associated with age 10–14 years (OR 2.38, 95% CI 1.15–4.93, *p* = 0.02), HIV positivity (OR 3.35, 95% CI 1.41–7.92, *p* = 0.01), hospitalization (OR 4.14, 95% CI 2.08–8.25, *p* < 0.001), and living outside of Kampala (OR 2.64, 95% CI 1.47–4.71, *p* = 0.001). Death was associated with hospitalization (OR 4.57, 95% CI 2.0–10.46, *p* < 0.001), severe malnutrition (OR 2.98, 95% CI 1.07–8.27, *p* = 0.04), baseline hepatomegaly (OR 4.11, 95% CI 2.09–8.09, *p* < 0.001), and living outside of Kampala (OR 2.41, 95% CI 1.17–4.96, *p* = 0.02).

**Conclusions:**

Empiric treatment of child TB was effective and safe, but treatment success remained below the 90% target. Addressing co-morbidities and improving retention in care may reduce unfavorable outcomes.

## Background

Timely initiation of treatment is critical for effective tuberculosis (TB) care and control in children. Of the estimated 233,000 children that die from TB each year, 96% did not receive treatment [1, 2]. For primary care providers in TB endemic settings, challenges with confirming a TB diagnosis in children and concern of adverse effects with empiric treatment (i.e., without microbiological confirmation) are key barriers to initiating anti-TB treatment [3, 4]. Symptoms are non-specific, chest x-ray findings variable, and diagnostic testing is often unavailable or not feasible due to difficulty obtaining sputum [3, 5]. Even when diagnostic testing occurs, sensitivity is decreased due to the paucibacillary nature of pediatric TB [6]. This contributes to 55% of child TB cases not being reported to national programs [2] from high TB burden settings have consistently documented that health care workers feel uncomfortable about making a TB diagnosis in a child and that there are often delays in care [7–9]. For example, a cross-sectional study of six primary clinics in Kampala found that no children who met clinical criteria for TB had been started on anti-TB treatment [10]. While algorithms exist to identify children with TB based on clinical factors [11–13], a concern is that without microbiologic confirmation, misdiagnosis could result in poor outcomes or adverse events from anti-TB treatment. A better understanding of outcomes of children treated empirically for TB based on a clinical diagnosis would provide more guidance to providers about the effectiveness and safety. We thus analyzed outcomes of children less than 15 years of age with clinically diagnosed TB receiving empiric treatment over a five-year period at a national referral hospital in Kampala, Uganda, and factors associated with the unfavorable outcomes of lost to follow up and death.

## Methods

### Study design and setting

This was a retrospective cohort study of children treated for clinically diagnosed TB at Mulago National Referral Hospital Pediatric TB Clinic. The clinic treats children up to 14 years of age with drug-susceptible TB using Fixed Dose Combination (FDC) treatment in accordance with Uganda National TB and Leprosy Programme (NTLP) guidelines [13]. Children are seen in clinic every two weeks during the first month of treatment and monthly thereafter for the standard six-month or twelve-month treatment depending on the TB disease type. At each visit, clinical notes are documented using standardized forms and entered into a secure electronic database. Adverse events are monitored based on clinical symptoms and exam, without routine laboratory testing. Management of any adverse events were completed according to national guidelines [12]. The Mulago Hospital Research and Ethics Committee approved the study protocol and waived the requirement for informed consent.

### Study population

We included all children less than 15 years old treated empirically for TB between January 2010 and December 2015. Clinical diagnosis was made per NTLP guidelines [12, 13]. We excluded children if they had multi-drug resistant (MDR) TB, were transferred, or were missing key variables (age and treatment outcome). As a comparison, outcomes on children with confirmed TB were also collected.

### Data collection and definitions

The data was not publically available hence we obtained Mulago Hospital permission to use the data. We extracted demographic and clinical data including HIV status, type of TB, TB treatment outcome, and any adverse events from the Pediatric TB Clinic electronic database. We defined severe malnutrition as a weight-for-age Z score less than − 3. We defined a favorable outcome if the child completed treatment and had documented clinical improvement, and an unfavorable outcome if the child died, failed treatment (no clinical improvement or treatment discontinuation) or was lost to follow-up prior to completion of treatment.

### Statistical analysis

Descriptive statistics were assessed with proportions and 95% confidence intervals (CI) for categorical variables and median and interquartile range (IQR) for continuous variables. For bivariate analysis of characteristics associated with favorable versus unfavorable outcome, we compared proportions using the chi-squared test. We stratified unfavorable outcomes into loss to follow up and death, and conducted logistic regression on characteristics with *p*-value < 0.05 from the unfavorable bivariate analysis. HIV status and severe malnutrition (known to be associated with unfavorable outcome) [14, 15], and sex were also included. Odds Ratios (OR) were presented with 95% Confidence Intervals (CI); *p*-value < 0.05 was considered significant. We performed analyses using STATA 15 (Stata Corp, College Station, TX, USA).

## Results

### Cohort characteristics

**Fig. 1:**
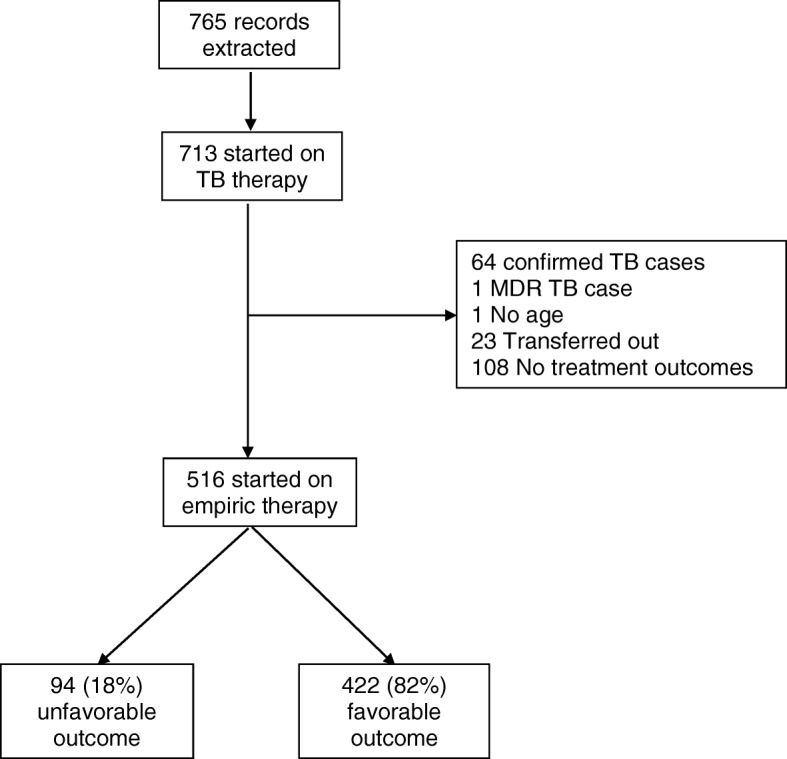
Participant Flowchart

Of 713 children treated for TB during the study period, there were 64 with confirmed TB, 1 with MDR TB, 1 with missing age information, 23 who were transferred out, and 108 with missing treatment outcome (Fig. 1), for a total of 516 children empirically started on anti-TB treatment.

Details of patient characteristics by outcome are shown in Table 1. The median age was 36 months (IQR 15–73), 55% (95% CI 51–60%) were male, and about half resided outside of Kampala district (46, 95% CI 41–50%). The majority (65, 95% CI 61–69%) were below five years old, and HIV prevalence was 6% (31/509, 95% CI 4–9%). The prevalence of severe malnutrition was 22% (76/349, 95% CI 18–26%). Over two-thirds (69%, 354/515, 95% CI 65–73%) of diagnoses were pulmonary TB cases.

**Table 1:** Demographic and clinical characteristics of children empirically treated for TB at Mulago Pediatric TB unit (*N* = 516)^*^

Characteristic	Favorable (*N* = 422)	Unfavorable (*N* = 94)	*p*-value^†^
	N (%, 95 CI)	N (%, 95 CI)	
Age group			
< 5 years	280 (66, 62–71)	57 (61,50–70)	0.006
5–9 years	104 (25, 21–29)	18 (19, 12–28)	
10–14 years	38 (9, 7–12)	19 (20, 13–30)	
Male Sex	238 (56, 52–61)	48 (51, 41–61)	0.35
Reside outside of Kampala	174 (41, 37–46)	62 (66, 56–75)	< 0.001
Pulmonary TB (*n* = 515)	291 (69, 65–73)	53 (67, 57–76)	0.69
HIV positive (*n* = 509)	19 (5, 3–7)	12 (14, 8–23)	0.001
Severe Malnutrition^‡^ (*n* = 349)	60 (20, 16–25)	16 (32, 20–46)	0.06
Hospitalized (*n* = 480)	33 (8, 6–11)	26 (34, 24–45)	< 0.001
Abnormal Chest X-ray (*n* = 367)	297 (93, 90–96)	46 (94, 82–98)	1.0
BCG vaccinated (*n* = 301)	239 (92, 88–95)	35 (83, 68–92)	0.06
Baseline Hepatomegaly (*n* = 383)	28 (8, 6–12)	13 (30, 18–46)	< 0.001
TST Positive (*n* = 246)	161 (75, 69–80)	18 (58, 40–74)	0.05

*N = 516 unless missing data, with number available indicated in parentheses

† *p*-value by Chi-squared or Fisher’s exact testing

‡ Severe malnutrition defined as weight-for-age Z score less than − 3

### Outcomes of empiric treatment

Of the 516 children empirically started on anti-TB treatment, 422 (82%) children had a favorable treatment outcome. Of the 94 children with unfavorable outcomes, 61% (57/94) were lost to follow-up, 37% (35/94) died, and 2% (2/94) failed treatment. The majority of deaths were in children under five years (22/35, 63%); three deaths occurred in children infected with HIV. The proportion of children on empiric treatment with favorable outcomes was similar to the children who had confirmed TB (48 of 54 with outcomes, 89%, versus 82% on empiric treatment, *p* = 0.17).

In total, there were eight drug reactions reported at follow up visits, with complaints that included paresthesia in the lower limbs, scaling in the plantar foot, vomiting, pallor and myalgia. However, there were no adverse events reported that required treatment discontinuation.

Unfavorable outcomes were more likely among children aged 10–14 years, HIV positive, not residing in Kampala, with baseline hepatomegaly, and hospitalization (Table 1). When stratified by loss to follow up and death (Table 2), loss to follow up was associated with age 10–14 years (OR 2.38, 95% CI 1.15–4.93, *p* = 0.02), HIV infection (OR 3.35, 95% CI 1.41–7.92, *p* = 0.01), hospitalization (OR 4.14, 95% CI 2.08–8.25, *p* < 0.001), and residing outside of Kampala (OR 2.64, 95% CI 1.47–4.71, *p* = 0.001). Death was associated with being hospitalized (OR 4.57, 95% CI 2.0–10.46, *p* < 0.001), severe malnutrition (OR 2.98, 95% CI 1.07–8.27, *p* = 0.04), hepatomegaly at baseline (OR 4.11, 95% CI 2.09–8.09, p < 0.001), and residing outside of Kampala (OR 2.41, 95% CI 1.17–4.96, *p* = 0.02).

**Table 2:** Factors associated with loss to follow up or death in children empirically treated at Mulago Pediatric TB unit, 2010–2015

	Loss to Follow Up		Death	
	OR (95% CI)	p-value	OR (95% CI)	*p*-value
Age Group				
< 5 yrs	REF	–	REF	–
5–9 yrs	0.88 (0.43–1.80)	0.73	0.87 (0.36–2.09)	0.76
10–14 yrs	2.38 (1.15–4.93)	0.02	1.68 (0.65–4.36)	0.28
Male sex	0.75 (0.43–1.31)	0.31	0.84 (0.42–1.67)	0.62
HIV positive	3.35 (1.41–7.92)	0.01	1.60 (0.46–5.57)	0.46
Hospitalization	4.14 (2.08–8.25)	< 0.001	4.57 (2.0–10.46)	< 0.001
resides outside of Kampala	2.64 (1.47–4.71)	0.001	2.41 (1.17–4.96)	0.02
Severe Malnutrition	1.46 (0.65–3.3)	0.36	2.98 (1.07–8.27)	0.04
Baseline Hepatomegaly	1.36 (0.83–2.22)	0.22	4.11 (2.09–8.09)	< 0.001

*CI* Confidence Interval, *OR* odds ratio, *HIV* Human Immunodeficiency Virus

## Discussion

In this study, we describe empiric treatment outcomes of children clinically diagnosed with TB at a referral center in Kampala, Uganda from 2010 to 2015. The majority of children (82%) had a favorable outcome with clinical improvement. However, this is below the World Health Organization (WHO) target of 90% treatment success [16]. There were no adverse events that required discontinuation of treatment. Loss to follow-up was the most common unfavorable outcome and was associated with older age (10–14 years), HIV infection, hospitalization, and living outside of Kampala. Death was the next cause of unfavorable outcome, and was associated with hospitalization, severe malnutrition, baseline hepatomegaly, and living outside of Kampala. Thus, empiric treatment was found to be safe and effective for most children, but greater efforts are needed to improve outcomes.

While the proportion with favorable outcome was below the global target, it corresponds with child TB outcomes in similar settings [16]. Retrospective studies in Nigeria, South Africa and Ethiopia found child TB (confirmed and clinically diagnosed) success rates of 77.4% [17], 78% [18], and 85.5% [19, 20], respectively. We also found a similar favorable proportion among the confirmed child TB cases. Our data support that empiric treatment of TB in children without microbiologic confirmation does not lead to worse outcomes.

Loss to follow-up was the most common reason for an unfavorable outcome. HIV co-infection has been associated with loss to follow up in youth [21, 22], and may be related to the additional pill burden, stigma and fear of discrimination. Hospitalization and not living in Kampala also were associated with loss to follow up; improved efforts are needed to ensure follow up after a child is discharged from the hospital, and to address barriers to obtaining care if the child does not live near the clinic. For example, Defeat TB is an initiative in Uganda to improve TB treatment success through health system strengthening to improve coordination of care and decentralize TB diagnosis and management [23].

Children age 10–14 years were more than twice as likely to be lost to follow up as children under 5 years. Compared to younger children, adolescents with TB face unique challenges to their care, including greater peer pressure, stigma, behavioral issues, substance abuse and prevalence of co-morbidities including HIV [2]. A retrospective analysis in South Africa found that 15% of adolescents with HIV aged 15–19 years discontinued TB treatment [24]. A study in Botswana found that adolescents were twice as likely to be lost to follow-up compared to adult [21]. These results emphasize that adolescent-friendly TB programs are needed to address their unique issues and continue to engage adolescents in care.

Mortality was the second most common unfavorable outcome. Our overall mortality was high at 7%, similar to the study in Nigeria that found a child TB mortality of 6% [17]. Consistent with past studies [17–19], our analyses found that factors related to more severe disease were associated with death, namely hospitalization, severe malnutrition and hepatomegaly. Living outside of Kampala district was also associated with death, and may reflect socioeconomic factors or delays in diagnosis and management.

Prior assessments of child TB outcomes have included both confirmed and clinically diagnosed TB. Confirmed cases may reflect children with higher bacterial load or occur in settings where there is greater access to diagnostics. By focusing only on children empirically treated for TB based on a clinical diagnosis, we sought to provide outcome data that was more generalizable to the majority of health care workers in low-resource settings.

There were also some potential limitations to our study. As a retrospective cohort, we cannot comment on the accuracy of the clinical diagnoses, although documented symptoms and signs were consistent with national guidelines. The study was conducted at a referral hospital, and may lower generalizability to primary care clinics. Mortality from alternative diagnoses were possible, and the exact causes of death were unknown. Adherence was not consistently documented. There was no available data on contacts with multi-drug resistant (MDR) TB to address empiric MDR treatment. The sample had a low proportion with HIV infection and limits the assessment of outcomes of empiric TB treatment in HIV-TB co-infected children. In particular, HIV-related mortality was low, and may be an underestimate as we did not include children with confirmed TB, who were transferred out or who had missing outcomes. The low number of unfavorable outcomes did not provide the power for a multivariable stratified regression analysis. Data was also not available on any follow-up laboratory testing to determine sub-clinical adverse events such as elevated liver transaminases on treatment.

## Conclusions

Our results highlight that initiation of empiric TB care in children is overall safe and effective if health care workers use appropriate clinical guidelines. However, they also suggest that diagnosis and initiation of treatment are only the beginning of the care cascade; health facility strengthening and age-appropriate care is critical to ensure favorable outcomes and retention in care.

## Abbreviations

aOR: Adjusted odds ratio
BCG: Bacillus Calmette–Guérin
CI: Confidence interval
FDC: Fixed Dose Combination
HIV: Human Immunodeficiency Virus
IQR: Interquartile Range
MDR: Multi-drug resistant
OR: Odds ratio
TB: Tuberculosis
TST: Tuberculin Skin Test
WHO: World Health Organization
